# MicroRNA and Degradome Profiling Uncover Defense Response of *Fraxinus velutina* Torr. to Salt Stress

**DOI:** 10.3389/fpls.2022.847853

**Published:** 2022-04-01

**Authors:** Jian Ning Liu, Xinmei Ma, Liping Yan, Qiang Liang, Hongcheng Fang, Changxi Wang, Yuhui Dong, Zejia Chai, Rui Zhou, Yan Bao, Lichang Wang, Shasha Gai, Xinya Lang, Ke Qiang Yang, Rong Chen, Dejun Wu

**Affiliations:** ^1^College of Forestry, Shandong Agricultural University, Tai’an, China; ^2^Shandong Provincial Academy of Forestry, Jinan, China; ^3^Shandong Taishan Forest Ecosystem Research Station, Shandong Agricultural University, Tai’an, China; ^4^State Forestry and Grassland Administration Key Laboratory of Silviculture in Downstream Areas of the Yellow River, Shandong Agricultural University, Tai’an, China; ^5^Culaishan Forest Farm, Tai’an, China

**Keywords:** *Fraxinus velutina* Torr., salt stress, microRNA, degradome, defense response

## Abstract

Soil salinization is a major environmental problem that seriously threatens the sustainable development of regional ecosystems and local economies. *Fraxinus velutina* Torr. is an excellent salt-tolerant tree species, which is widely planted in the saline-alkaline soils in China. A growing body of evidence shows that microRNAs (miRNAs) play important roles in the defense response of plants to salt stress; however, how miRNAs in *F. velutina* exert anti-salt stress remains unclear. We previously identified two contrasting *F. velutina* cuttings clones, salt-tolerant (R7) and salt-sensitive (S4) and found that R7 exhibits higher salt tolerance than S4. To identify salt-responsive miRNAs and their target genes, the leaves and roots of R7 and S4 exposed to salt stress were subjected to miRNA and degradome sequencing analysis. The results showed that compared with S4, R7 showed 89 and 138 differentially expressed miRNAs in leaves and roots, respectively. Specifically, in R7 leaves, miR164d, miR171b/c, miR396a, and miR160g targeting *NAC1*, *SCL22*, *GRF1*, and *ARF18*, respectively, were involved in salt tolerance. In R7 roots, miR396a, miR156a/b, miR8175, miR319a/d, and miR393a targeting *TGA2.3*, *SBP14*, *GR-RBP*, *TCP2/4*, and *TIR1*, respectively, participated in salt stress responses. Taken together, the findings presented here revealed the key regulatory network of miRNAs in R7 responding to salt stress, thereby providing new insights into improving salt tolerance of *F. velutina* through miRNA manipulation.

## Introduction

Soil salinization is a major environmental problem. It is estimated that by the year 2050, more than half of global arable land will be saline contamination (Butcher et al., 2016). Salinized soils hinder the growth and development of plants, resulting in the loss of biomass production, and even the deterioration of regional ecosystem (Polle and Chen, 2015; Hossain and Dietz, 2016; Ondrasek and Rengel, 2021). What’s more serious is that increasing soil salinization is now threatening sustainable development of local economies (Chen and Mueller, 2018). How to protect and restore the fragile ecosystem in salinized areas has become an urgent global issue. The selection and cultivation of naturally salt-tolerant plants are currently considered as an economically feasible strategy for the problem (Litalien and Zeeb, 2020). *Fraxinus velutina* Torr. is an excellent salt-tolerant tree species, which is widely planted in the saline-alkaline soils in Yellow River Delta, China (Mao et al., 2017). However, the mechanisms underlying salt tolerance of *F. velutina* remain largely unclear.

MicroRNAs (miRNAs) are a class of endogenous small non-coding RNAs with 18–25 nucleotides (nt), playing key regulatory roles in the defense response of plants to salt stress by regulating their target genes at a post-transcriptional level (Kumar et al., 2018; Song S. et al., 2019; Xu et al., 2021). The overexpression of miR528 in rice (*Oryza sativa*) can increase the contents of ascorbic and abscisic acid, and decrease reactive oxygen species (ROS) accumulation, thereby enhancing rice salt tolerance (Wang et al., 2021). Constitutive expression of rice miR528 and miR396 in creeping bentgrass modulates the growth and development, and enhances the response to salinity stress (Yuan et al., 2015, 2019). In wheat (*Triticum aestivum*), miRNA408 has been found to act as a crucial mediator in the tolerance to Pi deprivation and salt stress through modulating multiple stresses related to physiological processes (Bai et al., 2018). In maize (*Zea mays*), it is evidenced that *miR169/NF-YA* module is a key regulatory mediator in the response to salt stress in leaves and roots (Luan et al., 2014, 2015). In *Arabidopsis*, it is confirmed that miRNA393-associated regulatory modules can enhance the salt stress resistance by mediating several biological processes, including auxin signaling, redox-related components, osmoregulation and increased Na^+^ exclusion (Iglesias et al., 2014; Chen Z. et al., 2015; Denver and Ullah, 2019). The overexpressed miR414c in cotton (*Gossypium hirsutum*) can negatively regulate iron superoxide dismutase gene, thereby enhancing plant tolerance to salinity stress (Wang et al., 2019). A *miR156/SPL* regulatory module increases tolerance to salinity stress via up-regulating *MdWRKY100* in apple (*Malus domestica*) (Ma et al., 2021). However, little information is available about the regulatory role of miRNAs in *F. velutina* responding to salt stress.

As important regulatory molecules, plant miRNAs bind to their target mRNAs and regulate gene expression by direct cleavage and degradation of their targets (Djami-Tchatchou et al., 2017). To date, multiple approaches to study miRNAs, their target genes, and regulatory networks have been established (Sun et al., 2014). Of these approaches, the most commonly used is direct miRNA cloning and/or deep sequencing. Various experimental approaches like RLM-RACE and degradome sequencing have been developed to identify miRNA targets in plants (Shamimuzzaman and Vodkin, 2012; Wang C. et al., 2013). Degradome sequencing is a powerful approach that integrates a modified RLM-RACE and deep sequencing, which has been used to confirm miRNA targets globally in plants. Recently, the combination of miRNA and degradome sequencing has been widely used to identify the salt-responsive miRNAs and their target genes involved in regulating plant tolerance to salt stress (Yu et al., 2016; Kumar et al., 2017; Cervera-Seco et al., 2019; Zhang Y. et al., 2020; Xu et al., 2021).

In our previous study, we identified two contrasting cutting clones R7 (salt-tolerant) and S4 (salt-sensitive) of *F. velutina* in which R7 exhibits higher salt tolerance than S4. Meanwhile, we performed a comparative transcriptome analysis between R7 and S4, and identified some key genes and signaling pathways underlying high salt tolerance of R7 (Ma et al., 2022). In this study, to identify the salt-responsive miRNAs and their target genes involved in salt stress tolerance, an integration of miRNA and degradome sequencing analysis was performed on the leaves and roots of R7 and S4 with or without salt treatment. Our work revealed the key regulatory network of miRNAs in R7 responding to salt stress, thereby providing new insights into improving salt tolerance of *F. velutina* through miRNA manipulation.

## Materials and Methods

### Plant Materials and Culture Conditions

The salt-tolerant R7 and salt-sensitive S4 cutting clones of *F. velutina* were collected from the Experimental Base of Afforestation on Saline-Alkali Soil of Shandong Provincial Academy of Forestry, Shouguang city, China (118°42′9.18′′ E, 37°9′38.94′′ N). As previously described (Ma et al., 2022), 1-year-old cutting clones of both R7 and S4 were used in this study. After gently being removed rhizosphere soils, the clones were first pre-cultured in distilled water without any nutrient for 2 weeks, and then were transferred to a plastic container with 6 L of half-strength Hoagland’s solution for 4 weeks; the solution was refreshed every 7 days. All the experimental cutting clones were cultured in a growth incubator (LICHEN, Shanghai, China) with 25/20°C (day/night temperature), and 16 h light (1,200 μmol m^–2^ s^–1^)/8 h dark.

### Salt Treatment and Sampling

After 6 weeks of acclimatization, the healthy clones with uniform size were selected and treated with half-strength Hoagland’s solution containing 250 mM NaCl for 12 h; the seedlings without NaCl treatment were considered as the control. After treatment, the leaves and roots of R7 and S4 were collected and stored at −80°C for further analysis. The symbols of leaves and roots samples were letters ‘L’ and ‘R,’ respectively. For instance, the samples R7SL and R7CL represented the leaves of R7 cutting clones with or without salt treatment, respectively.

### Small RNA and Degradome Library Construction and Sequencing

Total of 24 samples (roots and leaves of R7 and S4, and two treatments with three biological replicates) were used small RNA and degradome library construction and sequencing. Total RNAs including small and large-size of tested samples were extracted using E.Z.N.A. Micro RNA Kit (Omega Bio-Tek, Norcross, GA, United States) following the manufacturer’s instructions. The quality and quantity of the isolated RNA were evaluated using a Bioanalyzer 2100 instrument (Agilent, Santa Clara, CA, United States) and an RNA 6000 Nano LabChip Kit (Agilent, Santa Clara, CA, United States), ensuring that the RNA integrity number value was higher than 8.0. For small RNA sequencing, the library of each sample was constructed using TruSeq Small RNA Library Preparation Kit (Illumina, San Diego, CA, United States), according to the manufacturer’s protocol. For degradome sequencing, two libraries from equally pooled samples of S7 and S4 were, respectively, constructed as previously described (German et al., 2008, 2009). Both small RNA and degradome libraries were sequenced on an Illumina Hiseq 4000 instrument (LC-Bio, Hangzhou, China) at single-end (50 bp).

### Identification of miRNAs

The raw reads were first trimmed using Trimmomatic v.0.39 (Bolger et al., 2014) to remove the junk reads, adapters, and low-complexity sequences. The validated reads were subjected to remove 3′ adaptor (5′-TGGAATTCTCGGGTGCCAAGG-3′) and perform length filter using Cutadapt v.3.5 (Kechin et al., 2017). The sequences with 18–25 nt were then subjected to exclude mRNA and other non-coding RNAs (tRNAs, rRNAs, snoRNAs, and snRNAs) via aligning the sequences, respectively to *F. velutina* mRNA sequences^1^ (Kelly et al., 2020) and Rfam v.14.6 database^2^ using the BLAST tool (BLASTN) (Altschul et al., 1990). The remaining unique sequences were subsequently aligned to the available plant miRNAs sequences stored in miRBase v.22.1^3^ to identify the conserved miRNAs in *F. velutina* using bowtie v1.3.0 (Langmead et al., 2009) with a maximum allowed mismatch ≤ 2.

The mature miRNA sequences obtained were mapped to the *F. velutina* reference genome sequence (see Text Footnote 1) by bowtie software, and then the corresponding flanking sequences of these miRNAs were extracted and used to predict the hairpin RNA structures using UNAfold v3.8 (Markham and Zuker, 2008). The potential miRNA precursors must meet the criteria as following: (1) the miRNAs were located on the arms (3′ or 5′) of stem-loop hairpin structure; (2) the miRNAs cannot contain large loops or breaks; (3) a maximum mismatches ≤ 6 were allowed between the miRNAs and their opposite sequences; (4) the predicted miRNA precursor structures must have minimal free folding energy index (>0.8) and negative minimal folding free energy, to differentiate them from other RNAs (Zhang et al., 2006; Yin et al., 2008).

The remaining small RNA sequences were used to identify novel miRNAs. After mapped to the *F. velutina* reference genome sequence, the flanking sequences of small RNAs were retrieved to predict secondary structures as the above mentioned. Only the small RNAs exhibiting a perfect stem-loop structure and meeting the criteria for plant miRNAs were identified as candidate novel miRNAs (Meyers et al., 2008).

### Degradome Sequencing Data Analysis and miRNA Targets Identification

After trimmed by Trimmomatic software, the validated degradome sequencing reads were subjected to predict the putative miRNA cleaved targets using the CleaveLand pipeline v.3.0 (Addo-Quaye et al., 2009). In brief, the sequencing reads were mapped to the *F. velutina* mRNA sequences downloaded from Ash Tree Genomes Database (see Text Footnote 1), and the perfect matching alignments were retained. The resulting tags with a 35–36 nt extended signature by adding 15 nt of upstream sequence were subsequently aligned to the identified mature miRNAs in this study, with a maximum allowed mismatch ≤ 5. Alignments where the degradome tag position coincided with the 10th or 11th nt of a given miRNA were kept and scored (Allen et al., 2005). According to the previously described, targets were classified into I, II, or III (Addo-Quaye et al., 2008). In addition, t-plots showing the distribution of signatures of miRNA cleaved targets were built using R package.

### Identification of Salt-Responsive miRNAs and Their Targets

The expression levels of the miRNAs were determined and normalized by transcripts per kilobase million. The differentially expressed miRNAs (DEmiRs) between each group were identified using R package DEseq2 (Love et al., 2014), with | log_2_ fold change| ≥ 1 and *P-*value ≤ 0.05.

We previously performed a comparative transcriptome analysis between R7 and S4, and identified some key genes and signaling pathways underlying high salt tolerance of R7 (Ma et al., 2022). To narrow the targets of salt-responsive miRNAs, our previous transcriptome data were used. For each comparison, the miRNA-target pairs containing DEmiRs and differentially expressed genes (DEGs) were selected for further analysis, and for each selected pair, the expression patterns of DEmiR were opposite to that of DEGs.

### Validation of the Identified miRNAs by Quantitative Real-Time PCR

To verify the results of small RNA sequencing, 15 DEmiRs including 12 conserved (ptc-miR160a, ptc-miR160g, mtr-miR164d, stu-MIR167d-p3_2ss6TC19CT, bna-MIR169c-p5_2ss12GC17TG, gma-miR169j-5p, mtr-MIR2592bj-p3_2ss12TC19AT, mtr-MIR2592bj-p3_2ss12TC19AT, mtr-miR393a_L+1, hbr-miR396a_R-1_2ss19TC20CT, hbr-miR396a_R-1_2ss19TC20CT, and gma-miR403a_R-1) and 3 novels (PC-3p-46517_176, PC-3p-56802_134, and PC-5p-55954_137) were randomly selected to perform quantitative real-time PCR (qRT-PCR) analysis (Supplementary Table 1). Total RNA isolated from leaves and roots using the E.Z.N.A. Micro RNA Kit (Omega Bio-Tek, Norcross, GA, United States) according to the manufacturer’s protocol. The primers were designed using Primer 5.0 software and synthesized by Sangon Biotech Co., Ltd. (Shanhai, China). The reverse transcription of miRNA was performed using Mir-X miRNA First-Strand Synthesis and TB Green qRT-PCR User Manual (Takara, Dalian, China) according to the manufacturer’s protocol.

The qRT-PCR was performed with the TB Green Premix Ex Taq II kit (Takara, Dalian, China) on a CFX Connect Real-Time instrument (Bio-Rad, Hercules, CA, United States). Each sample had three independent replicates. The 5.8s rRNA was used as an internal reference gene. The relative expression level of each miRNA was calculated based on the 2^–Δ^ ^Δ^ *^Ct^* method (Livak and Schmittgen, 2001).

### Statistical Analyses

Statistical data were presented as mean ± standard deviation (SD). Student’s *t*-test was used to compare the differences between two groups. A *P-*value ≤ 0.05 was considered to be significant difference. GraphPad Prism v.9.0 (GraphPad Software Inc., La Jolla, CA, United States) was used to perform statistical analysis.

## Results

### Identification of Conserved and Novel miRNAs in *F. velutina*

To identify miRNAs in *F. velutina* responding to salt stress, 24 small RNA sequencing libraries from leaf and root samples of R7 and S4 under salt treatment and control conditions were constructed and sequenced. In total, an average of 11.92 million raw reads for each sample were obtained. After removing low quality reads and adaptor sequences, approximately 2.13 million unique validated reads were obtained for each sample (Supplementary Table 2). After further excluding mRNA, tRNAs, rRNAs, snoRNAs, and snRNAs, an average of 2.12 million unique reads with length of 18–25 nt were generated for each sample (Supplementary Table 3).

**FIGURE 1:**
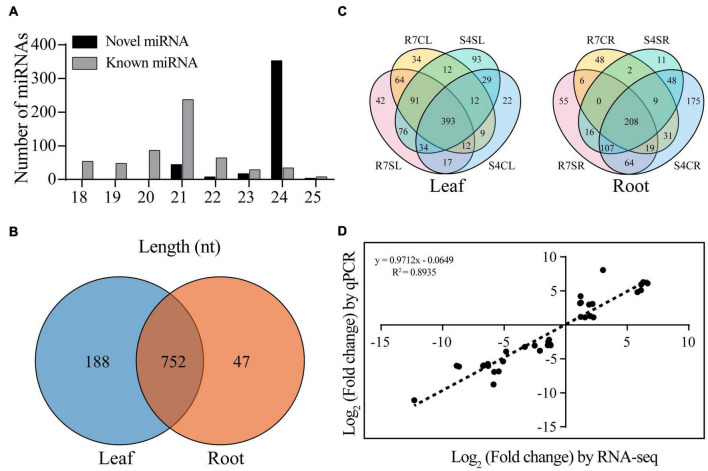
Identification of miRNAs from *Fraxinus velutina* tolerant (R7) and sensitive (S4) leaves and roots. **(A)** Length distribution of all miRNAs identified in this study. **(B)** The distribution of all miRNAs between leaf and root samples. **(C)** The distribution of expressed miRNAs of leaves and roots in R7 and S4 with or without salt treatment. **(D)** The correlation between small RNA sequencing (*x*-axis) and qRT-PCR (*y*-axis) results. R7SL, R7 leaves with salt treatment; R7CL, R7 leaves without salt treatment; R7SR, R7 roots with salt treatment; R7CR, R7 roots without salt treatment; S4SL, S4 leaves with salt treatment; S4CL, S4 leaves without salt treatment; S4SR, S4 roots with salt treatment; S4CR, S4 roots without salt treatment.

To identify the conserved and novel miRNAs in *F. velutina*, the filtered unique reads were aligned to the miRBase v.22.1 database and *F. velutina* reference genome. A total of 987 miRNAs including 560 conserved and 427 novel miRNAs were identified. The majority of miRNAs were distributed between 20 and 24 nt, with 24 nt exhibiting the highest abundance (Figure 1A). On the basis of sequence similarity, these miRNAs were further classified into 50 miRNA families, with MIR156, MIR159, MIR166, and MIR396 presenting relatively high abundance (Supplementary Figure 1A and Supplementary Table 4).

### Identification of Specifically Expressed miRNAs Between Salt-Tolerant R7 and Salt-Sensitive S4

Based on the normalized expression levels, the correlation analysis between the expression levels within each sample was analyzed. The results showed that the correlation coefficient, γ^2^ between three biological replicates in each group was 0.878, indicating the replicates were highly consistent (Supplementary Figure 1B). There were 188 and 47 specifically expressed miRNAs in leaf and root, respectively, and 752 shared miRNAs in both tissues (Figure 1B). There were 796 and 800 miRNAs were expressed in R7 and S4 leaves, respectively; 42 and 93 miRNAs were exclusively expressed in R7 and S4 leaves under salt stress, respectively (Figure 1C). In roots, there were 565 and 690 miRNAs expressed in R7 and S4, respectively; 55 and 11 miRNAs were exclusively expressed in R7 and S4 roots under salt treatment, respectively (Figure 1C).

To confirm the small RNA sequencing data, 15 DEmiRs were randomly selected to conduct qRT-PCR analysis. The results showed that these selected miRNAs exhibited the same expression patterns with that of small RNA sequencing data (Supplementary Figure 2). In addition, based on the log_2_ fold change of each comparison, the correlation analysis between qRT-PCR results and sequencing data was performed. The results revealed a high correlation coefficient (*R*^2^ = 0.8935) between sequencing data and qRT-PCR results, demonstrating that the sequencing data are accurate and reliable (Figure 1D).

### Identification of Target Genes for miRNAs

**FIGURE 2:**
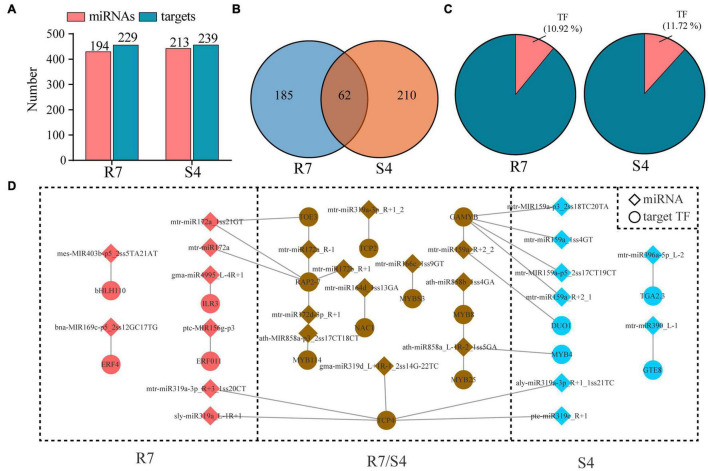
Prediction of miRNA targets based on degradome sequencing data. **(A)** Number of miRNAs and target genes in all miRNA-target pairs in *Fraxinus velutina* tolerant (R7) and sensitive (S4). **(B)** The distribution of miRNA-target pairs between R7 and S4. **(C)** The percentage of transcript factors (TFs) in all targets from R7 and S4. **(D)** Network of all miRNA-TF pairs in R7 and S4.

To investigate biological function of these miRNAs, degradome sequencing from R7 and S4 samples were performed to identify the putative target genes for the miRNAs. After trimmed and polished, 9719583 and 13021448 validated reads were generated from R7 and S4, respectively. The reads were further mapped to the *F. velutina* reference genome, and the results revealed that more than 99% of the reads were perfectly aligned back to the reference (Supplementary Table 5). After analyzing by CleaveL and, 247 miRNA-target pairs, including 194 miRNAs targeting 229 genes were identified in R7; 273 pairs, including 213 miRNAs targeting 239 genes were in S4 (Figure 2A and Supplementary Table 6). Among the miRNA-target pairs, 185 and 210 pairs were exclusively identified in R7 and S4, respectively; 62 pairs were overlapped in both R7 and S4 (Figure 2B). Among the targets, we found that there were 25 (10.92%) and 28 (11.72%) genes encoding transcription factors (TF) targeted by the miRNAs in R7 and S4, respectively (Figure 2C). For example, an ethylene-responsive transcription factor 4 (*ERF4*) was targeted by bna-MIR169c-p5_2ss12GC17TG in R7, and a transcription factor TGA2.3 (*TGA2.3*) was regulated by mtr-miR390_L-1 in S4. Additionally, we also found multiple shared miRNA-TF pairs in both plants, such as mtr-miR166c_1ss9GT-MYBS3 and mtr-miR164d_1ss13GA-NAC1 (Figure 2D).

### Identification of Salt Stress-Responsive miRNAs

**FIGURE 3:**
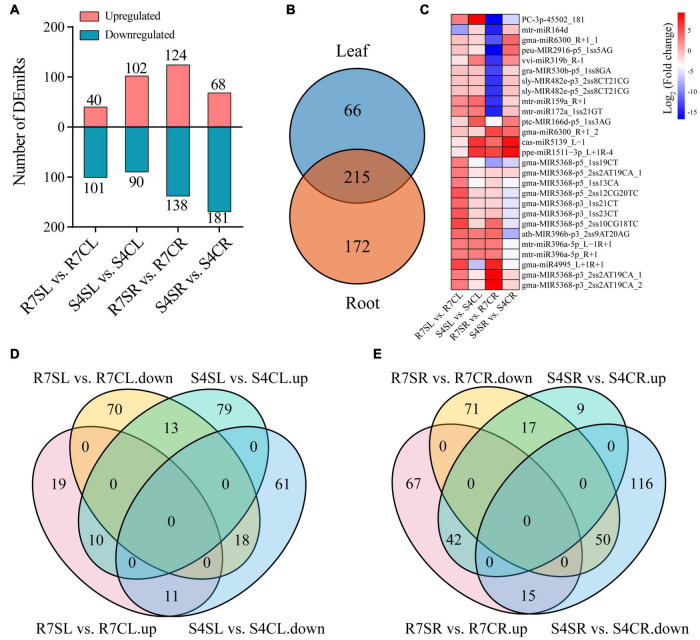
Identification of salt stress-responsive miRNAs. **(A)** Statistics of up- and downregulated differentially expressed miRNAs (DEmiRs) in leaves and roots. The red and green represented up- and down-regulated DEmiRs, respectively. **(B)** The distribution of DEmiRs between leaves and roots. **(C)** Expression patterns of the shared DEmiRs in all comparisons. From red to blue indicated the fold change from high to low. **(D)** The distribution of up- and downregulated DEmiR in R7 and S4 leaves. **(E)** The distribution of up- and down-regulated DEmiR in R7 and S4 roots. R7SL, R7 leaves with salt treatment; R7CL, R7 leaves without salt treatment; R7SR, R7 roots with salt treatment; R7CR, R7 roots without salt treatment; S4SL, S4 leaves with salt treatment; S4CL, S4 leaves without salt treatment; S4SR, S4 roots with salt treatment; S4CR, S4 roots without salt treatment.

To identify miRNAs involved in response to salt stress, the differentially expressed miRNAs (DEmiRs) was analyzed using DEseq2. The results revealed 141 (40 up- and 101 down-regulated miRNAs) and 192 (102 up- and 90 down-regulated miRNAs) DEmiRs in R7, and S4 leaves, respectively (Figure 3A). In roots, 262 DEmiRs including 124 up- and 138 down-regulated miRNAs were identified in R7, and 249 DEmiRs including 68 up- and 181 down-regulated miRNAs were in S4 (Figure 3A and Supplementary Table 7). Under salt stress, there were 66 and 172 specific DEmiRs in leaf and root samples, respectively, with 215 shared DEmiRs in both tissues (Figure 3B and Supplementary Table 7). Among these shared DEmiRs, 27 DEmiRs presented in all comparisons, in which only 8 DEmiRs showed the consistent or opposite expression patterns between these two tissues in two clones after salt treatment (Figure 3C). The distribution analysis of leaf DEmiRs revealed that 24 DEmiRs exhibited opposite expression patterns between R7 and S4 after salt treatment (Figure 3D). Among the root DEmiRs, 32 DEmiRs presented opposed regulations between R7 and S4 after salt stress (Figure 3E).

### Analysis of Salt Stress-Responsive miRNA Targets

**FIGURE 4:**
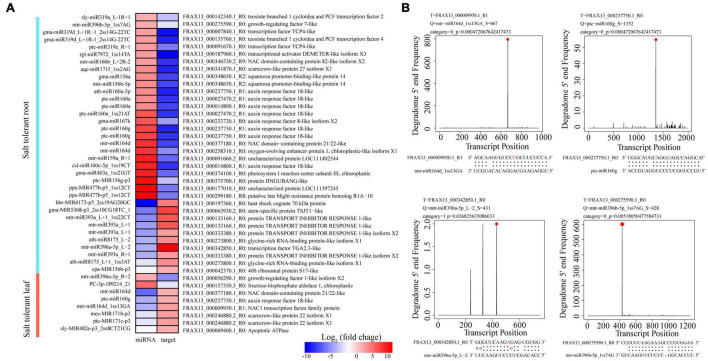
Analysis of salt stress-responsive miRNAs in *F. velutina* tolerant ‘R7’ leaves and roots. **(A)** Expression patterns of miRNAs and targets from specific DEmiR-DEG pairs in R7 leaves and roots. From red to blue represented the fold change from high to low. **(B)** Target plots (t-plots) of the selected DEmiR-DEG pairs in R7 leaves and roots confirmed by degradome sequencing. The red circle and letter represented slice site.

To explore regulatory roles of DEmiRs in *F. velutina* response to salt stress, our previous transcriptome data were used to narrow the miRNA-target pairs. The results showed that 65 DEmiR-DEG pairs were identified among the comparisons (Supplementary Table 8). To further investigate the mechanisms underlying enhanced salt tolerance in R7, the specific DEmiRs in R7 leaves (89 DEmiRs) and roots (138 DEmiRs) were further analyzed. In the leaves, 8 specific DEmiR-DEG pairs, including 8 DEmiRs targeting 7 DEGs were identified (Table 1). Among these pairs, multiple DEGs were related to the plant response to salt stress, such as mtr-miR164d_1ss13GA targeted NAC1 transcription factor (*NAC1*), ptc-miR160g targeted *ARF18* (Figures 4A,B). In the roots, there were 35 specific DEmiR-DEG pairs including 29 DEmiRs targeting 29 DEGs (Table 2). Among these pairs, multiple DEGs were associated with the plant response to salt stress, such as mtr-miR396a-5p_L-2 targeted *TGA2.3* and mtr-miR396b-5p_1ss7AG targeted growth-regulating factor 7 (*GRF7*) (Figures 4A,B).

**TABLE 1:** The specific DEmiR-DEG modules in *Fraxinus velutina* ‘R7’ leaf under salt stress.

miRNA name	miRNA sequence	R7SL/R7CL	Target transcript	R7SL/R7CL	Annotation	Gene symbol
mtr-miR164d.1	TGGAGAAGCAGGGCACATGCT	−8.86	FRAX13_000377180.1_R0	2.25	NAC domain-containing protein 21/22-like	NAC021
mtr-miR164d.2	TGGAGAAGCAGGACACATGCT	−5.42	FRAX13_000009950.1_R1	2.5	NAC1 transcription factor family protein	NAC1
ptc-miR160g	TGCCTGGCTCCCTGGATGCCA	−5.42	FRAX13_000237750.1_R0	2.33	Auxin response factor 18-like	ARF18
mes-MIR171b-p3	TGATTGAGCCGTGCCAATATC	−3.09	FRAX13_000246880.2_R0	2.95	Scarecrow-like protein 22	SCL22
ptc-MIR171c-p3	TTGAGCCGCGCCAATATCACT	−2.96	FRAX13_000246880.2_R0	2.95	Scarecrow-like protein 22	SCL22
sly-MIR482e-p3	TTTCCTATTCCTCCCATACCGA	−1.28	FRAX13_000069400.1_R0	2.02	Apoptotic ATPase	–
PC-3p-189214	AAGATTGCCCACTGTGGACAGGAG	4.91	FRAX13_000157350.3_R0	−2.59	Fructose-bisphosphate aldolase 1	FBA1
mtr-miR396a-5p	TTCCACAGCTTTCTTGAACTTTT	1.6	FRAX13_000056290.1_R0	−6.63	Growth-regulating factor 1-like	GRF1

**TABLE 2:** The specific DEmiR-DEG modules in *Fraxinus velutina* ‘R7’ root under salt stress.

miRNA name	miRNA sequence	R7SR/R7CR	Target transcript	R7SR/R7CR	Annotation	Gene symbol
aqc-miR171f	TGATTGAGCCGTGCCAATATC	3.28	FRAX13_000341870.1_R0	-12.02	Scarecrow protein 27	SCL27
ath-miR160a-5p	TGCCTGGCTCCCTGTATGCCA	3.96	FRAX13_000237750.1_R1	-11.55	Auxin response factor 18	ARF18
ath-miR8175.1	CGTTCCCCGGCAACGGCGCCA	–2.8	FRAX13_000273800.1_R0	3.55	Glycine-rich RNA-binding protein	GR-RBP
ath-miR8175.2	TCCCCGGCAACGGCGCCA	–5.62	FRAX13_000273800.1_R0	3.55	Glycine-rich RNA-binding protein	GR-RBP
cpa-MIR156b-p3	GCTCACTTCTCTTTCTGTCAGC	–1.91	FRAX13_000042370.1_R0	2.35	40S ribosomal protein S17	RibS17
csi-miR160c-5p	TGCCTGGCTCCCTGTATGTTT	8.4	FRAX13_000014800.1_R1	-10.82	Auxin response factor 18	ARF18
gma-miR156a	TGACAGAAGAGAGTGAGCAC	3.52	FRAX13_000348650.1_R2	-9.94	Squamosa promoter-binding protein 14	SBP14
gma-miR167k	TGAAGCTGCCAGCCTGATCTTA	6.25	FRAX13_000233720.1_R0	-6.15	Auxin response factor 8	ARF18
gma-miR319d	TTGGACTGAAGGGAGCTCCTC	2.38	FRAX13_000007840.1_R0	-13.08	Transcription factor TCP4	TCP4
gma-miR319d	TTGGACTGAAGGGAGCTCCTC	2.38	FRAX13_000135760.1_R0	-11.57	Transcription factor TCP4	TCP4
gma-miR403a	TTAGATTCACGCACAAACTTT	8.58	FRAX13_000374100.1_R0	-8.67	Photosystem I reaction center subunit III	PsaF
gma-MIR5368-p3	TGGGATTGGGTTTGGGCC	–7.99	FRAX13_000063930.2_R0	7.12	Stem-specific protein TSJT1	TSJT1
hbr-MIR6173-p5	GATACCCCAGTAGTCCTAGCC	–13.69	FRAX13_000197560.1_R0	4.33	Heat shock cognate 70 kDa protein	HSP70
mtr-miR156b-5p	TGACAGAAGAGAGTGAGCAC	3.52	FRAX13_000348650.1_R2	-9.94	Squamosa promoter-binding protein 14	SBP14
mtr-miR159a	TTTGGATTGAAGGGAGCTCTAA	8.11	FRAX13_000091660.2_R0	-8.75	Uncharacterized protein	-
mtr-miR164d	TGGAGAAGCAGGGCACATGCT	7.94	FRAX13_000377180.1_R0	-10.59	NAC domain-containing protein 21/22	NAC021
mtr-miR164d	TGGAGAAGCAGGGCACATGCT	7.94	FRAX13_000258310.1_R0	-6.67	Oxygen-evolving enhancer protein 1	PSBO1
mtr-miR166b	TCTCGGACCAGGCTTCATTCC	2.92	FRAX13_000346530.2_R9	-11.21	NAC domain-containing protein 82	NAC082
mtr-miR393a.1	TTCCAAAGGGATCGCATTGATC	–6.32	FRAX13_000133160.1_R0	6.07	TRANSPORT INHIBITOR RESPONSE 1	TIR1
mtr-miR393a.1	TTCCAAAGGGATCGCATTGATC	–6.32	FRAX13_000333380.1_R0	3.12	TRANSPORT INHIBITOR RESPONSE 1	TIR1
mtr-miR393a.2	TTCCAAAGGGATCGCATTGATT	–7.31	FRAX13_000133160.1_R0	6.07	TRANSPORT INHIBITOR RESPONSE 1	TIR1
mtr-miR393a.3	TCCAAAGGGATCGCATTGATCT	–5.01	FRAX13_000333380.1_R0	3.12	TRANSPORT INHIBITOR RESPONSE 1	TIR1
mtr-miR396a-5p.1	CCACAGCTTTCTTGAACTT	–5.52	FRAX13_000342850.1_R0	9.76	Transcription factor TGA2.3	TGA2.3
mtr-miR396b-5p.2	TTCCACGGCTTTCTTGAACTG	1.91	FRAX13_000275590.1_R0	-2.54	Growth-regulating factor 7	GRF14
ppe-MIR477b-p5	CCTCAAGGGCTTCCAATATTCC	10.53	FRAX13_000177010.1_R0	-7.99	Uncharacterized protein	-
ppe-MIR477b-p5	CCTCAAGGGCTTCCAATATTCC	10.53	FRAX13_000299180.1_R8	-7.76	Putative late blight resistance protein homolog R1A-10	R1A-10
ptc-MIR156g-p3	GCTCTCTAGTCTTCTGTCATC	9.29	FRAX13_000375700.1_R0	-4.34	Protein JINGUBANG	JGB
ptc-miR160a.1	TGCCTGGCTCCCTGTATGCCA	3.96	FRAX13_000027470.2_R1	-10.75	Auxin response factor 18	ARF18
ptc-miR160a.1	TGCCTGGCTCCCTGTATGCCA	3.96	FRAX13_000014800.1_R0	-10.49	Auxin response factor 18	ARF18
ptc-miR160a.2	TGCCTGGCTCCCTGTATGCCT	6.16	FRAX13_000027470.2_R1	-10.75	Auxin response factor 18	ARF18
ptc-miR160g	TGCCTGGCTCCCTGGATGCCA	6.66	FRAX13_000237750.1_R1	-11.55	Auxin response factor 18	ARF18
ptc-miR160g	TGCCTGGCTCCCTGGATGCCA	6.66	FRAX13_000237750.1_R0	-10.76	Auxin response factor 18	ARF18
ptc-miR319e	TTGGACTGAAGGGAGCTCCTC	2.38	FRAX13_000091670.1_R0	-8.21	Transcription factor TCP4	TCP4
rgl-miR7972	TTGTCAGGCTTGTAATTCTCC	2.67	FRAX13_000187960.1_R0	-13.96	Transcriptional activator DEMETER	DEM
sly-miR319a	TTGGACTGAAGGGAGCTCCT	1.68	FRAX13_000142340.1_R0	-4.74	Transcription factor TCP2	TCP2

## Discussion

In our previous study, we identified two *F. velutina* cuttings clones, salt-tolerant R7 and salt-sensitive S4 and found that R7 exhibits higher salt tolerance than S4. Meanwhile, we performed a comparative transcriptome analysis between R7 and S4, and identified several crucial genes and signaling pathways involved in high salt tolerance of R7 (Ma et al., 2022). In the present study, utilizing an integration of miRNA, mRNA, and degradome sequencing data analysis; we identified several key miRNA-target modules contributing to the high salt tolerance of R7. Specifically, the miRNA-target modules identified in R7 leaf were primarily related with antioxidant system and auxin signaling; while miRNA-target modules in R7 root mainly belonged to ion homeostasis and ROS scavenging.

It is well reported that *miR171/GRAS* module is an important contributor to plant development and biotic and abiotic stress resistance in *Medicago truncatula* (Hirsch et al., 2009). In apple, *miR171i/SCL26.1* module can enhance drought stress tolerance via regulating antioxidant system (Wang et al., 2020). In the present study, we found both miR171b (mes-MIR171b-p3) and miR171c (mes-MIR171b-p3) upregulated a GRAS transcription factor *SCL22* (FRAX13_000246880.2_R0), suggesting that *miR171b/c-SCL22* module is involved in regulating antioxidant system, resulting in the enhanced tolerance to salt stress. The *miR396a-5p/GRF1* module enhances tobacco (*Nicotiana tabacum*) tolerance to salt stress (Chen L. et al., 2015). Under stress, *GRF1* inhibits plant growth by regulating *WRKY28* expression in *Arabidopsis* (Piya et al., 2020). The present study found that miR396a (mtr-miR396a-5p) over-expression down-regulated *GRF1* (FRAX13_000056290.1_R0), further down-regulated *WRKY28*, implying that *miR396a/GRF1/WRKY28* module enhances tolerance to salt stress by promoting plant growth. Thus, *miR171b/c-SCL22* and *miR396a/GRF1/WRKY28* modules are important regulators in enhancing salt stress resistance in *F. velutina*.

Auxin response factors (ARFs) have been confirmed to play crucial roles in plant tolerance to abiotic stress through regulating the auxin signaling pathway (Liu et al., 2018; Song X. et al., 2019; Cui et al., 2020). In poplar, *miR390/TAS3/ARFs* module has been confirmed to be a key regulator of lateral root growth of poplar (*Populus* spp.) plants under salt stress by modulating the auxin pathway (He et al., 2018). In the present study, we found miR160g (ptc-miR160g) up-regulated *ARF18* (FRAX13_000237750.1_R0) expression, suggesting that the *miR160g/ARF18* module contributes to salt tolerance via modulating auxin pathway. Previous study has shown that the overexpressed miR393 regulates rice salt and drought tolerance by inhibiting transport inhibitor response protein (*TIR1*) (Xia et al., 2012). In this study, we found that miR393a (mtr-miR393a.1, mtr-miR393a.2, and mtr-miR393a.3) down-regulated *TIR1* (FRAX13_000333380.1_R0) under salt stress, indicating that *miR393a/TIR1* module enhances plant tolerance to salt stress via mediating auxin signaling pathway. Together, *miR160g/ARF18* and *miR393a/TIR1* modules enhance tolerance to salt stress by regulating auxin signaling pathway in *F. velutina*.

Much evidence shows that NAC transcription factors play vital roles in plant development (Guo et al., 2005; Petricka et al., 2012), cell apoptosis (Lee et al., 2014), and abiotic stress tolerance (Tran et al., 2010). Overexpressed *PeNAC1* in *Arabidopsis* enhances tolerance to salt stress by regulating Na*^+^*/K*^+^* homeostasis (Wang J. Y. et al., 2013). In this study, we found that miR164d (mtr-miR164d) targeted *NAC1* (FRAX13_000009950.1_R1) and up-regulated NAC1 expression in R7 subjected to salt stress, suggesting that *miR164d/NAC1* module enhances salt tolerance through regulating Na*^+^*/K*^+^* homeostasis. The increasing evidence has shown that the TGA (TGACG motif-binding factor) transcription factors, a basic leucine zipper (*bZIP*) gene subfamily, play crucial roles in response to salt stress (Du et al., 2014; Zhang et al., 2014). The overexpression of *GmTGA13* enhances *Arabidopsis* and soybean (*Glycine max*) salt tolerance through regulating ion homeostasis (Ke et al., 2021). In the present study, we found *TGA2.3* (FRAX13_000342850.1_R0) was targeted by miR396a (mtr-miR396a-5p.1), suggesting that *miR396a/TGA2.3* module enhances salt tolerance via mediating ion homeostasis. Thus, *miR164d/NAC1* and *miR396a/TGA2.3* modules contribute to the enhanced salt tolerance in *F. velutina*.

Multiple studies have shown that squamosa-promoter binding protein box (*SBP*-box) can regulate the salt tolerance in many plants, such as *Betula platyphylla* Suk (Ning et al., 2017) and rice (Lan et al., 2019). Silencing *CaSBP12* in pepper (*Capsicum annuum*) enhances tolerance to salt stress and reduces the ROS accumulation (Zhang H. X. et al., 2020). In this study, we found that miR156a/b (gma-miR156a and mtr-miR156b-5p) targeted *SBP14* (FRAX13_000348650.1_R2) and up-regulated *SBP14* expression under salt stress, suggesting that *miR156a/b-SBP14* module increases ROS scavenging, thereafter leading to the enhanced salt tolerance. A previous study has shown that GR-RBPs is a positive regulatory molecule in regulating ROS accumulation to enhance salt tolerance in *Arabidopsis* (Tan et al., 2014). Our study showed that miR8175 (ath-miR8175.1 and ath-miR8175.2) directly bonded to *GR-RBP* (FRAX13_000273800.1_R0) and up-regulated *GR-RBP* expression in R7 subjected to salt stress, indicating that *miR8175/GR-RBP* module enhances tolerance to salt stress. Collectively, *miR156a/b-SBP14* and *miR8175/GR-RBP* modules are key regulators, regulating ROS scavenging, of high salt tolerance in *F. velutina*.

## Conclusion

**FIGURE 5:**
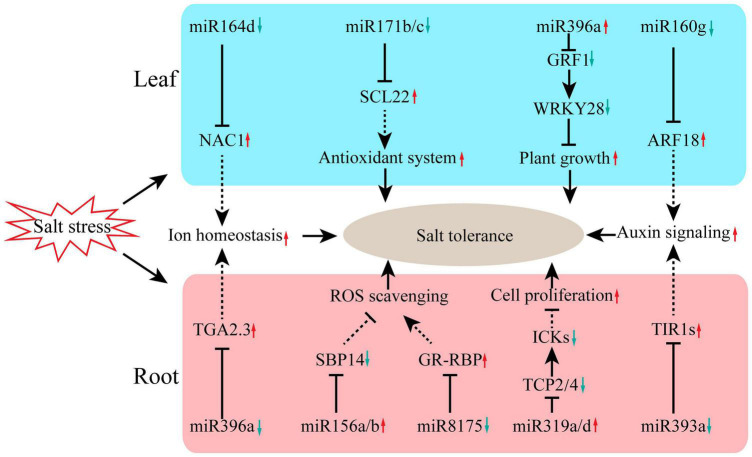
The miRNA-related molecular mechanisms for enhancing salt tolerance of *F. velutina* ‘R7’. Under salt stress, multiple miRNA/target modules were involved in R7 response to salt stress. In the leaf, miR164d/NAC1, miR171/SCL22, miR396a/GRF1/WRKY28, and miR160g/ARF18 modules were involved in plant response to salt stress by regulating multiple biological processes, such as antioxidant system and auxin signaling. In the root, miR396a/TGA2.3, miR156/SBP14, miR319/TCPs/ICKs, and miR393a/TIR1 modules enhanced plant tolerance to salt stress by regulating several processes including ROS scavenging, cell proliferation, and ion homeostasis.

In summary, this work revealed the key regulatory network of miRNAs in salt-tolerant clone R7 of *F. velutina* responding to salt stress. The small RNA and degradome sequencing data presented here allowed us to propose potential regulatory roles of miRNAs in the defense response of R7 to salt stress (Figure 5). Under salt stress, multiple miRNA/target modules were involved in R7 response to salt stress. In the leaf, *miR164d/NAC1*, *miR171/SCL22*, *miR396a/GRF1/WRKY28*, and *miR160g/ARF18* modules were involved in plant response to salt stress by regulating multiple biological processes, such as antioxidant system and auxin signaling. In the root, *miR396a/TGA2.3*, *miR156/SBP14*, *miR319/TCPs/ICKs*, and *miR393a/TIR1* modules enhanced plant tolerance to salt stress by regulating several processes including ROS scavenging, cell proliferation, and ion homeostasis. The miRNA-target modules identified here pave a novel avenue for improving the salt tolerance of *F. velutina* through miRNA manipulation.

## Data Availability Statement

The original contributions presented in the study are publicly available. This data can be found here: The small RNA and degradome sequencing data presented in this study can be available at the Sequence Read Archive under accession number PRJNA793056.

## Author Contributions

KY and DW conceptualized the research program. JL, XM, and LY finished the analysis of this study and wrote the manuscript. QL, HF, CW, and LW conducted the RNA sequencing data analysis. YD and ZC designed the qRT-PCR experiment and finished the operation. RZ, YB, XL, SG, and RC planted the material and finished the physiology analysis. KY, RC, and DW revised the manuscript. All authors discussed the results, commented on the manuscript, and approved the submitted version.

## Conflict of Interest

The authors declare that the research was conducted in the absence of any commercial or financial relationships that could be construed as a potential conflict of interest.

## Publisher’s Note

All claims expressed in this article are solely those of the authors and do not necessarily represent those of their affiliated organizations, or those of the publisher, the editors and the reviewers. Any product that may be evaluated in this article, or claim that may be made by its manufacturer, is not guaranteed or endorsed by the publisher.

## Footnotes

1
http://ashgenome.org/

2
http://rfam.xfam.org/

3
http://www.mirbase.org/

## Supplementary Material

Supplementary Figure 1 Analysis of miRNA sequencing data. **(A)** Number of conserved miRNAs in each family. **(B)** Heatmap showed the correlation coefficient (*r*^2^) between each sample.

Supplementary Figure 2 The qRT-PCR analysis was performed to verify the reliability of miRNA sequencing data. The heatmap showed the log_2_ fold change of 15 selected miRNAs in different comparisons of qRT-PCR results and sequencing data. From blue to red represented the value of log2 fold change from low to high.

Supplementary Table 1 All primers used in this study.

Supplementary Table 2 Summary of the small RNA sequences from each sample.

Supplementary Table 3 Statistics of the small RNAs identified in each sample.

Supplementary Table 4 The details of all miRNAs identified in this study.

Supplementary Table 5 The overview of the degradome sequencing data.

Supplementary Table 6 Identification of target genes of the miRNAs.

Supplementary Table 7 Identification of the differentially expressed miRNAs.

Supplementary Table 8 Identification of target genes of the differentially expressed miRNAs.

## References

[B1] Addo-Quaye C. Eshoo T. W. Bartel D. P. Axtell M. J. (2008). Endogenous siRNA and miRNA targets identified by sequencing of the *Arabidopsis* degradome. *Curr. Biol.* 18 758–762. 10.1016/j.cub.2008.04.042 18472421 PMC2583427

[B2] Addo-Quaye C. Miller W. Axtell M. J. (2009). CleaveLand: a pipeline for using degradome data to find cleaved small RNA targets. *Bioinformatics* 25 130–131. 10.1093/bioinformatics/btn604 19017659 PMC3202307

[B3] Allen E. Xie Z. Gustafson A. M. Carrington J. C. (2005). microRNA-directed phasing during trans-acting siRNA biogenesis in plants. *Cell* 121 207–221. 10.1016/j.cell.2005.04.004 15851028

[B4] Altschul S. F. Gish W. Miller W. Myers E. W. Lipman D. J. (1990). Basic local alignment search tool. *J. Mol. Biol.* 215 403–410. 10.1016/s0022-2836(05)80360-2 2231712

[B5] Bai Q. Wang X. Chen X. Shi G. Liu Z. Guo C. (2018). Wheat miRNA taemiR408 acts as an essential mediator in plant tolerance to Pi deprivation and salt stress via modulating stress-associated physiological processes. *Front. Plant Sci.* 9:499. 10.3389/fpls.2018.00499 29720988 PMC5916090

[B6] Bolger A. M. Lohse M. Usadel B. (2014). Trimmomatic: a flexible trimmer for Illumina sequence data. *Bioinformatics* 30 2114–2120. 10.1093/bioinformatics/btu170 24695404 PMC4103590

[B7] Butcher K. Wick A. F. DeSutter T. Chatterjee A. Harmon J. (2016). Soil salinity: a threat to global food security. *Agron. J.* 108 2189–2200. 10.2134/agronj2016.06.0368

[B8] Cervera-Seco L. Marques M. A. C. Sanz-Carbonell A. Marquez-Molins J. Carbonell A. Darï S. J. (2019). Identification and characterization of stress-responsive TAS3-derived tasiRNAs in melon. *Plant Cell Physiol.* 60 2382–2393. 10.1093/pcp/pcz131 31290971

[B9] Chen J. Mueller V. (2018). Coastal climate change, soil salinity and human migration in Bangladesh. *Nat. Clim. Chang.* 8 981–985. 10.1038/s41558-018-0313-8

[B10] Chen L. Luan Y. Zhai J. (2015). Sp-miR396a-5p acts as a stress-responsive genes regulator by conferring tolerance to abiotic stresses and susceptibility to phytophthora nicotianae infection in transgenic tobacco. *Plant Cell Rep.* 34 2013–2025. 10.1007/s00299-015-1847-0 26242449

[B11] Chen Z. Hu L. Han N. Hu J. Yang Y. Xiang T. (2015). Overexpression of a miR393-resistant form of transport inhibitor response protein 1 (mTIR1) enhances salt tolerance by increased osmoregulation and Na^+^ exclusion in *Arabidopsis thaliana*. *Plant Cell Physiol.* 56 73–83. 10.1093/pcp/pcu149 25336111

[B12] Cui J. Li X. Li J. Wang C. Cheng D. Dai C. (2020). Genome-wide sequence identification and expression analysis of ARF family in sugar beet (*Beta vulgaris* L.) under salinity stresses. *PeerJ* 8:e9131. 10.7717/peerj.9131 32547857 PMC7276148

[B13] Denver J. B. Ullah H. (2019). miR393s regulate salt stress response pathway in *Arabidopsis thaliana* through scaffold protein RACK1A mediated ABA signaling pathways. *Plant Signal. Behav.* 14:1600394. 10.1080/15592324.2019.1600394 31021701 PMC6546147

[B14] Djami-Tchatchou A. T. Sanan-Mishra N. Ntushelo K. Dubery I. A. (2017). Functional roles of microRNAs in agronomically important plants-potential as targets for crop improvement and protection. *Front. Plant Sci.* 8:378. 10.3389/fpls.2017.00378 28382044 PMC5360763

[B15] Du X. Du B. Chen X. Zhang S. Zhang Z. Qu S. (2014). Overexpression of the MhTGA2 gene from crab apple (*Malus hupehensis*) confers increased tolerance to salt stress in transgenic apple (*Malus domestica*). *J. Agric. Sci.* 152 634–641. 10.1017/S0021859613000130

[B16] German M. A. Luo S. Schroth G. Meyers B. C. Green P. J. (2009). Construction of parallel analysis of RNA ends (PARE) libraries for the study of cleaved miRNA targets and the RNA degradome. *Nat. Protoc.* 4 356–362. 10.1038/nprot.2009.8 19247285

[B17] German M. A. Pillay M. Jeong D. H. Hetawal A. Luo S. Janardhanan P. (2008). Global identification of microRNA-target RNA pairs by parallel analysis of RNA ends. *Nat. Biotechnol.* 26 941–946. 10.1038/nbt1417 18542052

[B18] Guo H. S. Xie Q. Fei J. F. Chua N. H. (2005). MicroRNA directs mRNA cleavage of the transcription factor NAC1 to downregulate auxin signals for arabidopsis lateral root development. *Plant Cell* 17 1376–1386. 10.1105/tpc.105.030841 15829603 PMC1091761

[B19] He F. Xu C. Fu X. Shen Y. Guo L. Leng M. (2018). The microRNA390/trans-acting short interfering RNA3 module mediates lateral root growth under salt stress via the auxin pathway. *Plant Physiol.* 177 775–791. 10.1104/pp.17.01559 29717017 PMC6001319

[B20] Hirsch S. Kim J. Muñoz A. Heckmann A. B. Downie J. A. Oldroyd G. E. (2009). GRAS proteins form a DNA binding complex to induce gene expression during nodulation signaling in *Medicago truncatula*. *Plant Cell* 21 545–557. 10.1105/tpc.108.064501 19252081 PMC2660633

[B21] Hossain M. S. Dietz K. J. (2016). Tuning of redox regulatory mechanisms, reactive oxygen species and redox homeostasis under salinity stress. *Front. Plant Sci.* 7:548. 10.3389/fpls.2016.00548 27242807 PMC4861717

[B22] Iglesias M. J. Terrile M. C. Windels D. Lombardo M. C. Bartoli C. G. Vazquez F. (2014). MiR393 regulation of auxin signaling and redox-related components during acclimation to salinity in *Arabidopsis*. *PLoS One* 9:e107678. 10.1371/journal.pone.0107678 25222737 PMC4164656

[B23] Ke D. He Y. Fan L. Niu R. Cheng L. Wang L. (2021). The soybean TGA transcription factor GmTGA13 plays important roles in the response to salinity stress. *Plant Biol. (Stuttg)* 24 313–322. 10.1111/plb.13360 34741387

[B24] Kechin A. Boyarskikh U. Kel A. Filipenko M. (2017). cutPrimers: a new tool for accurate cutting of primers from reads of targeted next generation sequencing. *J. Comput. Biol.* 24 1138–1143. 10.1089/cmb.2017.0096 28715235

[B25] Kelly L. J. Plumb W. J. Carey D. W. Mason M. E. Cooper E. D. Crowther W. (2020). Convergent molecular evolution among ash species resistant to the emerald ash borer. *Nat. Ecol. Evol.* 4 1116–1128. 10.1038/s41559-020-1209-3 32451426 PMC7610378

[B26] Kumar D. Dutta S. Singh D. Prabhu K. V. Kumar M. Mukhopadhyay K. (2017). Uncovering leaf rust responsive miRNAs in wheat (*Triticum aestivum* L.) using high-throughput sequencing and prediction of their targets through degradome analysis. *Planta* 245 161–182. 10.1007/s00425-016-2600-9 27699487

[B27] Kumar V. Khare T. Shriram V. Wani S. H. (2018). Plant small RNAs: the essential epigenetic regulators of gene expression for salt-stress responses and tolerance. *Plant Cell Rep.* 37 61–75. 10.1007/s00299-017-2210-4 28951953

[B28] Lan T. Zheng Y. Su Z. Yu S. Song H. Zheng X. (2019). OsSPL10, a SBP-box gene, plays a dual role in salt tolerance and trichome formation in rice (*Oryza sativa* L.). *G3 (Bethesda)* 9 4107–4114. 10.1534/g3.119.400700 31611344 PMC6893181

[B29] Langmead B. Trapnell C. Pop M. Salzberg S. L. (2009). Ultrafast and memory-efficient alignment of short DNA sequences to the human genome. *Genome Biol.* 10:R25. 10.1186/gb-2009-10-3-r25 19261174 PMC2690996

[B30] Lee S. Lee H. J. Huh S. U. Paek K. H. Ha J. H. Park C. M. (2014). The *Arabidopsis* NAC transcription factor NTL4 participates in a positive feedback loop that induces programmed cell death under heat stress conditions. *Plant Sci.* 227 76–83. 10.1016/j.plantsci.2014.07.003 25219309

[B31] Litalien A. Zeeb B. (2020). Curing the earth: a review of anthropogenic soil salinization and plant-based strategies for sustainable mitigation. *Sci. Total Environ.* 698:134235. 10.1016/j.scitotenv.2019.134235 31783465

[B32] Liu N. Dong L. Deng X. Liu D. Liu Y. Li M. (2018). Genome-wide identification, molecular evolution, and expression analysis of auxin response factor (ARF) gene family in *Brachypodium distachyon* L. *BMC Plant Biol.* 18:336. 10.1186/s12870-018-1559-z 30522432 PMC6282295

[B33] Livak K. J. Schmittgen T. D. (2001). Analysis of relative gene expression data using real-time quantitative PCR and the 2(-Delta Delta C(T)) Method. *Methods* 25 402–408. 10.1006/meth.2001.1262 11846609

[B34] Love M. I. Huber W. Anders S. (2014). Moderated estimation of fold change and dispersion for RNA-seq data with DESeq2. *Genome Biol.* 15:550. 10.1186/s13059-014-0550-8 25516281 PMC4302049

[B35] Luan M. Xu M. Lu Y. Zhang L. Fan Y. Wang L. (2015). Expression of zma-miR169 miRNAs and their target ZmNF-YA genes in response to abiotic stress in maize leaves. *Gene* 555 178–185. 10.1016/j.gene.2014.11.001 25445264

[B36] Luan M. Xu M. Lu Y. Zhang Q. Zhang L. Zhang C. (2014). Family-wide survey of miR169s and NF-YAs and their expression profiles response to abiotic stress in maize roots. *PLoS One* 9:e91369. 10.1371/journal.pone.0091369 24633051 PMC3954700

[B37] Ma X. Liu J. N. Yan L. Liang Q. Fang H. Wang C. (2022). Comparative transcriptome analysis unravels defense pathways of *Fraxinus velutina* Torr against salt stress. *Front. Plant Sci.* 2022:842726. 10.3389/fpls.2022.842726 35310642 PMC8931533

[B38] Ma Y. Xue H. Zhang F. Jiang Q. Yang S. Yue P. (2021). The miR156/SPL module regulates apple salt stress tolerance by activating *MdWRKY100* expression. *Plant Biotechnol. J.* 19 311–323. 10.1111/pbi.13464 32885918 PMC7868983

[B39] Mao P. Tang Q. Cao B. Liu J. Shao H. Cao Z. (2017). Eco-physiological adaptability in mixtures of *Robinia pseudoacacia* and *Fraxinus velutina* and coastal eco-engineering. *Ecol. Eng.* 106 109–115. 10.1016/j.ecoleng.2017.05.021

[B40] Markham N. R. Zuker M. (2008). UNAFold: software for nucleic acid folding and hybridization. *Methods Mol. Biol.* 453 3–31. 10.1007/978-1-60327-429-6_1 18712296

[B41] Meyers B. C. Axtell M. J. Bartel B. Bartel D. P. Baulcombe D. Bowman J. L. (2008). Criteria for annotation of plant microRNAs. *Plant Cell* 20 3186–3190. 10.1105/tpc.108.064311 19074682 PMC2630443

[B42] Ning K. Chen S. Huang H. Jiang J. Yuan H. Li H. (2017). Molecular characterization and expression analysis of the SPL gene family with BpSPL9 transgenic lines found to confer tolerance to abiotic stress in *Betula platyphylla* Suk. *Plant Cell Tissue Organ. Cult. (PCTOC)* 130 469–481. 10.1007/s11240-017-1226-3

[B43] Ondrasek G. Rengel Z. (2021). Environmental salinization processes: detection, implications & solutions. *Sci. Total Environ.* 754 142432. 10.1016/j.scitotenv.2020.142432 33254867

[B44] Petricka J. J. Winter C. M. Benfey P. N. (2012). Control of *Arabidopsis* root development. *Annu. Rev. Plant Biol.* 63 563–590. 10.1146/annurev-arplant-042811-105501 22404466 PMC3646660

[B45] Piya S. Liu J. Burch-Smith T. Baum T. J. Hewezi T. (2020). A role for *Arabidopsis* growth-regulating factors 1 and 3 in growth-stress antagonism. *J. Exp. Bot.* 71 1402–1417. 10.1093/jxb/erz502 31701146 PMC7031083

[B46] Polle A. Chen S. (2015). On the salty side of life: molecular, physiological and anatomical adaptation and acclimation of trees to extreme habitats. *Plant Cell Environ.* 38 1794–1816. 10.1111/pce.12440 25159181

[B47] Shamimuzzaman M. Vodkin L. (2012). Identification of soybean seed developmental stage-specific and tissue-specific miRNA targets by degradome sequencing. *BMC Genomics* 13:310. 10.1186/1471-2164-13-310 22799740 PMC3410764

[B48] Song S. Hao L. Zhao P. Xu Y. Zhong N. Zhang H. (2019). Genome-wide identification, expression profiling and evolutionary analysis of auxin response factor gene family in potato (*Solanum tuberosum* Group Phureja). *Sci. Rep.* 9:1755. 10.1038/s41598-018-37923-7 30742001 PMC6370904

[B49] Song X. Li Y. Cao X. Qi Y. (2019). MicroRNAs and their regulatory roles in plant-environment interactions. *Annu. Rev. Plant Biol.* 70 489–525. 10.1146/annurev-arplant-050718-100334 30848930

[B50] Sun X. Zhang Y. Zhu X. Korir N. K. Tao R. Wang C. (2014). Advances in identification and validation of plant microRNAs and their target genes. *Physiol. Plant* 152 203–218. 10.1111/ppl.12191 24641625

[B51] Tan Y. Qin Y. Li Y. Li M. Ma F. (2014). Overexpression of MpGR-RBP1, a glycine-rich RNA-binding protein gene from *Malus prunifolia* (Willd.) Borkh., confers salt stress tolerance and protects against oxidative stress in *Arabidopsis*. *Plant Cell Tissue Organ. Cult. (PCTOC)* 119 635–646. 10.1007/s11240-014-0563-8

[B52] Tran L. S. Nishiyama R. Yamaguchi-Shinozaki K. Shinozaki K. (2010). Potential utilization of NAC transcription factors to enhance abiotic stress tolerance in plants by biotechnological approach. *GM Crops* 1 32–39. 10.4161/gmcr.1.1.10569 21912210

[B53] Wang C. Han J. Korir N. K. Wang X. Liu H. Li X. (2013). Characterization of target mRNAs for grapevine microRNAs with an integrated strategy of modified RLM-RACE, newly developed PPM-RACE and qPCRs. *J. Plant Physiol.* 170 943–957. 10.1016/j.jplph.2013.02.005 23582890

[B54] Wang J. Y. Wang J. P. He Y. (2013). A *Populus euphratica* NAC protein regulating Na+/K+ homeostasis improves salt tolerance in *Arabidopsis thaliana*. *Gene* 521 265–273. 10.1016/j.gene.2013.03.068 23545306

[B55] Wang M. Guo W. Li J. Pan X. Pan L. Zhao J. (2021). The miR528-AO module confers enhanced salt tolerance in rice by modulating the ascorbic acid and abscisic acid metabolism and ROS scavenging. *J. Agric. Food Chem.* 69 8634–8648. 10.1021/acs.jafc.1c01096 34339211

[B56] Wang W. Liu D. Chen D. Cheng Y. Zhang X. Song L. (2019). MicroRNA414c affects salt tolerance of cotton by regulating reactive oxygen species metabolism under salinity stress. *RNA Biol.* 16 362–375. 10.1080/15476286.2019.1574163 30676211 PMC6380294

[B57] Wang Y. Feng C. Zhai Z. Peng X. Wang Y. Sun Y. (2020). The apple microR171i-SCARECROW-LIKE PROTEINS26.1 module enhances drought stress tolerance by integrating ascorbic acid metabolism. *Plant Physiol.* 184 194–211. 10.1104/pp.20.00476 32680976 PMC7479918

[B58] Xia K. Wang R. Ou X. Fang Z. Tian C. Duan J. (2012). OsTIR1 and OsAFB2 downregulation via OsmiR393 overexpression leads to more tillers, early flowering and less tolerance to salt and drought in rice. *PLoS One* 7:e30039. 10.1371/journal.pone.0030039 22253868 PMC3254625

[B59] Xu T. Zhang L. Yang Z. Wei Y. Dong T. (2021). Identification and functional characterization of plant miRNA under salt stress shed light on salinity resistance improvement through miRNA manipulation in crops. *Front. Plant Sci.* 12:665439. 10.3389/fpls.2021.665439 34220888 PMC8247772

[B60] Yin Z. Li C. Han X. Shen F. (2008). Identification of conserved microRNAs and their target genes in tomato (*Lycopersicon esculentum*). *Gene* 414 60–66. 10.1016/j.gene.2008.02.007 18387754

[B61] Yu Y. Wu G. Yuan H. Cheng L. Zhao D. Huang W. (2016). Identification and characterization of miRNAs and targets in flax (*Linum usitatissimum*) under saline, alkaline, and saline-alkaline stresses. *BMC Plant Biol.* 16:124. 10.1186/s12870-016-0808-2 27234464 PMC4884397

[B62] Yuan S. Li Z. Li D. Yuan N. Hu Q. Luo H. (2015). Constitutive expression of rice microRNA528 alters plant development and enhances tolerance to salinity stress and nitrogen starvation in creeping bentgrass. *Plant Physiol.* 169 576–593. 10.1104/pp.15.00899 26224802 PMC4577425

[B63] Yuan S. Zhao J. Li Z. Hu Q. Yuan N. Zhou M. (2019). MicroRNA396-mediated alteration in plant development and salinity stress response in creeping bentgrass. *Hortic. Res.* 6:48. 10.1038/s41438-019-0130-x 31069081 PMC6491569

[B64] Zhang B. H. Pan X. P. Cox S. B. Cobb G. P. Anderson T. A. (2006). Evidence that miRNAs are different from other RNAs. *Cell Mol. Life Sci.* 63 246–254. 10.1007/s00018-005-5467-7 16395542 PMC11136112

[B65] Zhang H. X. Zhu W. C. Feng X. H. Jin J. H. Wei A. M. Gong Z. H. (2020). Transcription factor CaSBP12 negatively regulates salt stress tolerance in pepper (*Capsicum annuum* L.). *Int. J. Mol. Sci.* 21:444. 10.3390/ijms21020444 31936712 PMC7013666

[B66] Zhang J. Y. Qu S. C. Qiao Y. S. Zhang Z. Guo Z. R. (2014). Overexpression of the *Malus hupehensis* MhNPR1 gene increased tolerance to salt and osmotic stress in transgenic tobacco. *Mol. Biol. Rep.* 41 1553–1561. 10.1007/s11033-013-3001-9 24407603

[B67] Zhang Y. Gong H. Li D. Zhou R. Zhao F. Zhang X. (2020). Integrated small RNA and degradome sequencing provide insights into salt tolerance in sesame (*Sesamum indicum* L.). *BMC Genomics* 21:494. 10.1186/s12864-020-06913-3 32682396 PMC7368703

