# Supplementary figures and images for: MicroRNA and Degradome Profiling Uncover Defense Response of Fraxinus velutina Torr. to Salt Stress
Source: Front Plant Sci. 2022 Apr 1;13:847853. doi: 10.3389/fpls.2022.847853 (PMC9011107; doi:10.3389/fpls.2022.847853)

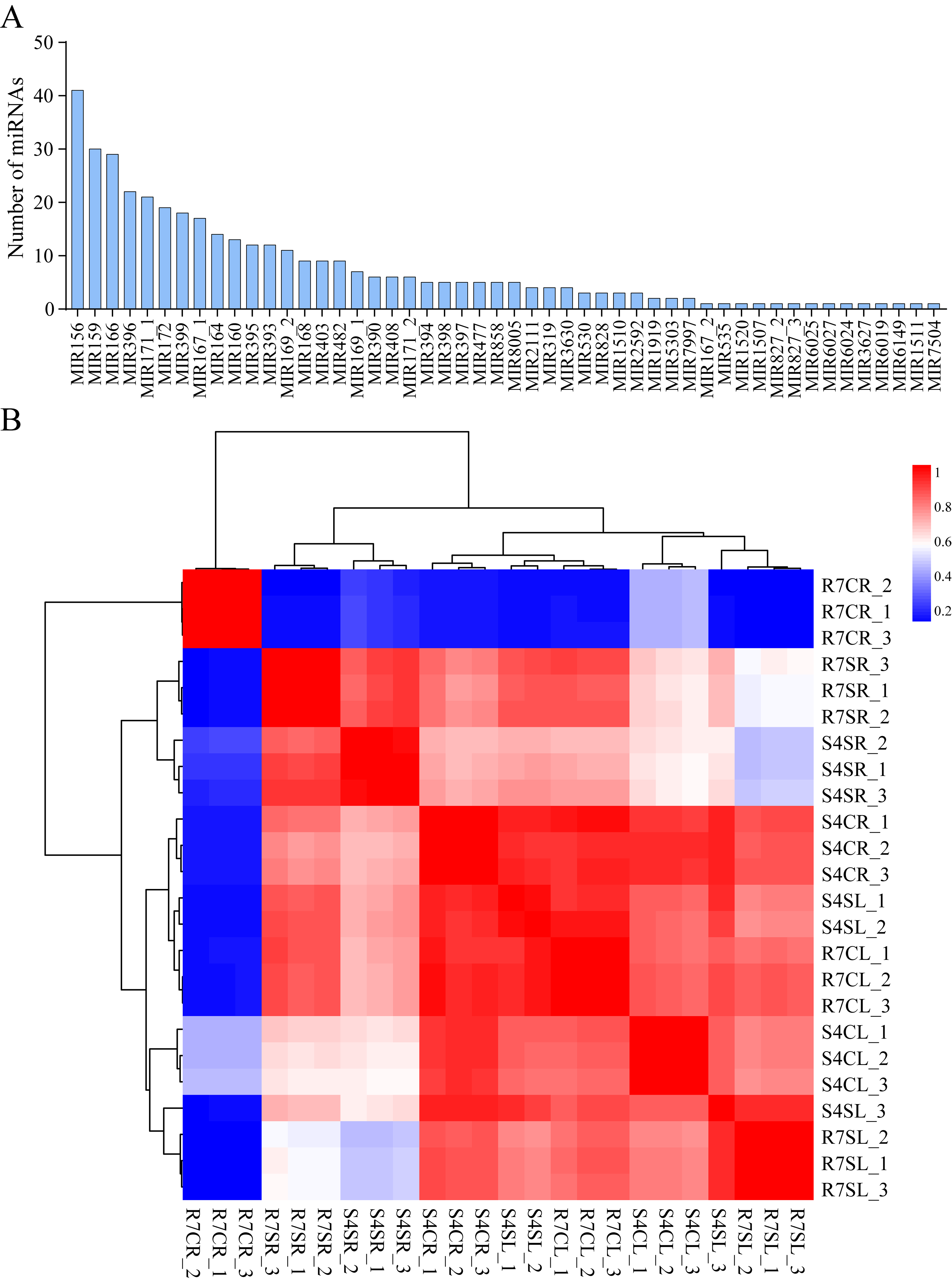

Supplement: Supplementary Figure 1 — Analysis of miRNA sequencing data. (A) Number of conserved miRNAs in each family. (B) Heatmap showed the correlation coefficient (r2) between each sample. [file Image_1.TIF]

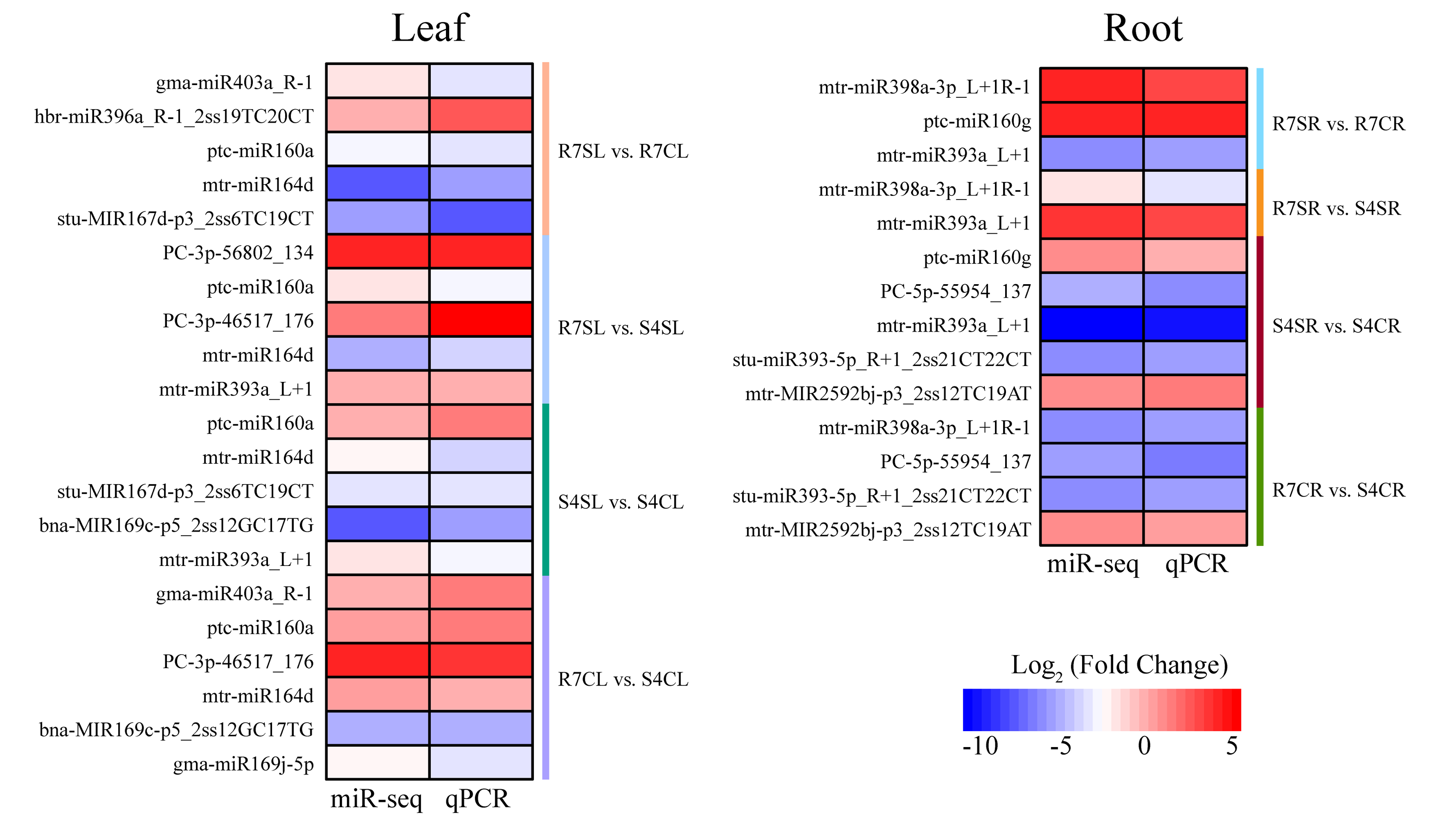

Supplement: Supplementary Figure 2 — The qRT-PCR analysis was performed to verify the reliability of miRNA sequencing data. The heatmap showed the log2 fold change of 15 selected miRNAs in different comparisons of qRT-PCR results and sequencing data. From blue to red represented the value of log2 fold change from low to high. [file Image_2.TIF]
